# Characterization of Argonaute Nuclease from Mesophilic Bacterium *Chroococcidiopsis*

**DOI:** 10.3390/ijms26031085

**Published:** 2025-01-27

**Authors:** Yanhong Peng, Yue Zhang, Yang Liu, Lixin Ma

**Affiliations:** State Key Laboratory of Biocatalysis and Enzyme Engineering, Hubei Key Laboratory of Industrial Biotechnology, School of Life Sciences, Hubei University, Wuhan 430062, China; pyhong@stu.hubu.edu.cn (Y.P.); zy990401@outlook.com (Y.Z.)

**Keywords:** mesophilic prokaryotic argonaute, *Chroococcidiopsis* sp., ChAgo, DNA cleavage

## Abstract

Mesophilic microbial sources of prokaryotic Argonaute (pAgo) programmable nucleases have garnered considerable attention for their potential applications in genome editing and molecular diagnostics. In this study, we characterized a novel pAgo from the mesophilic bacterium *Chroococcidiopsis* sp. (ChAgo), which can cleave single-stranded DNA (ssDNA) using both 5′-phosphorylated guide DNA (5′P-gDNA) and 5′-hydroxylated guide DNA (5′OH-gDNA). Efficient cleavage occurs using 14–25 nt 5′P-gDNA and 13–20 nt 5′OH-gDNA in the presence of Mn^2+^ ions at temperatures ranging from 25 to 75 °C, with optimal activity at 55 °C. ChAgo demonstrates low tolerance for single-base mismatches, similar to other pAgo proteins. The cleavage efficiency varies based on the guide/target pair, with mismatches at specific positions significantly reducing activity. For instance, mismatches at positions 4, 5, or 12 in T-gDNA/target pairs and at positions 5 or 8–10 in g38NT-gDNA/target pairs notably decrease efficiency. ChAgo’s sensitivity to mismatches makes it a promising tool for nucleic acid manipulation and detection, requiring initial screening for high cleavage efficiency sites and subsequent identification of mismatch positions.

## 1. Introduction

Argonaute (Ago) proteins are ubiquitous in archaea, bacteria, and eukaryotes, and are particularly well-known for their function as effector nucleases within the small RNA-mediated gene silencing machinery in eukaryotes [1,2,3]. Prokaryotic Agos (pAgos) and eukaryotic Agos (eAgos) exhibit distinct preferences, with pAgos possessing the ability to cleave RNA/DNA targets utilizing RNA/DNA as guides [3,4,5,6]. Similar to the clustered regularly interspaced short palindromic repeats (CRISPR)/CRISPR-associated (Cas) system, pAgos are members of the nucleic acid-guided microbial defense systems, safeguarding the host against the invasion of mobile genetic elements. Considering the significant impact of CRISPR on genome editing, there is growing interest in exploring the gene-editing capabilities of pAgos [7,8,9,10]. pAgos, derived from thermophilic bacteria and archaea, exhibit optimal activity temperatures exceeding 65 °C but display low endonuclease activity within the 20–37 °C range, making them impractical for genome editing applications in mesopilous organisms such as human beings [11,12,13,14]. The identification of mesophilic pAgos lays the groundwork for the development of pAgo-based biotechnology.

Recently, substantial progress has been achieved in the investigation of mesophilic pAgos [15,16,17,18,19]. *Natronobacterium gregoryi* Ago (NgAgo) has exhibited the capacity to modulate gene expression in mammalian cells using DNA as a guide, as well as selectively target specific genomic DNA loci to control gene transcription in zebrafish [20,21]. Furthermore, both NgAgo and *Clostridium butyricum* Ago (CbAgo) have demonstrated the ability to enhance homology-directed recombination in bacterial systems [9,10,22,23]. Nevertheless, mesophilic pAgos, in contrast to Cas proteins, lack the ability to unwind double-stranded DNA (dsDNA) and are currently incapable of achieving the same level of efficiency in genome cleavage as the CRISPR/Cas system [5,7,9,23,24]. Mesophilic pAgos have shown considerable potential for application in the field of molecular diagnosis. For example, *Kurthia massiliensis* Ago (KmAgo) can be integrated with reverse transcriptase at moderate temperatures to detect SARS-CoV-2 [25]. Furthermore, several biosensors utilizing CbAgo have been developed to detect pathogenic bacteria, nucleic acid targets, and non-nucleic acid targets at ambient temperature [26,27,28]. Further investigation is crucial to identify a wider range of special mesophilic pAgos and explore their potential practical applications.

In this study, we characterized a mesophilic pAgo from *Chroococcidiopsis* sp. (ChAgo), which can use ssDNAs as guides to specifically cleave both ssDNA targets and RNA targets at 37 °C. ChAgo demonstrates a low tolerance for mismatches between guide and target sequences, thereby ensuring its high specificity. These findings not only enhance our comprehension of pAgos, but also hold promise for advancing the development of pAgo-based technologies operating at moderate temperatures.

## 2. Results

### 2.1. Sequence Alignment and Phylogenetic Tree of ChAgo

**Figure 1:**
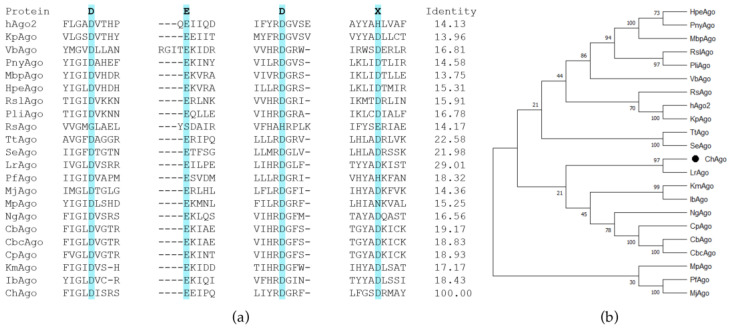
Sequence analysis and the visualization of phylogenetic trees. (**a**) Multiple sequence alignment of ChAgo with several other characterized Ago proteins. (**b**) A maximum likelihood phylogenetic analysis of ChAgo was conducted based on its amino acid sequences. The bootstrap values at the nodes represent the confidence levels from a maximum likelihood analysis based on 500 resampled data sets. ChAgo is denoted by a black circle.

As KmAgo was reported to be one of the pAgos most closely related to eAgos [4], we selected KmAgo (WP_010289662.1) as the query and utilized the BLASTp program to search for mesophilic pAgos. ChAgo from the cyanobacterium *Chroococcidiopsis* sp. was selected as a candidate due to the robust adaptability and wide ecological niche of this cyanobacterial species [29]. Furthermore, although phylogenetically closest to LrAgo from *Limnothrix rosea*, ChAgo exhibits only 29.01% sequence identity (Figure 1). A multiple sequence alignment of ChAgo with other Ago proteins (Figure 1a) revealed that ChAgo possesses the conserved DEDD (D533, E575, D600, and D721) catalytic residues crucial for nuclease activity in ‘slicing’ Ago proteins [30]. We generated catalytically inactive variants of ChAgo (ChAgo-DM) by substituting two of the four catalytic tetrad residues (D533A and D600A).

### 2.2. Single-Stranded Nucleic Acid Cleavage Assay

**Figure 2:**
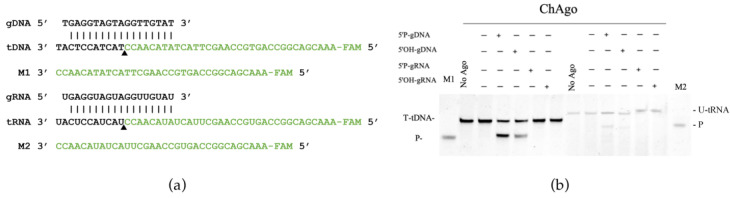
Analysis of the nucleic acid cleavage activity of ChAgo. (**a**) Sequences of the synthetic guide and target used in in vitro cleavage assays. The black triangle marks the cleavage site. Vertical lines represent contiguous Watson–Crick base pairing. (**b**) Cleavage of FAM-labeled DNA and RNA targets by ChAgo. M1, 34 nt long ssDNA; M2, 34 nt long RNA; P, cleavage products.

To investigate the biochemical properties of ChAgo, we successfully expressed both ChAgo and its mutant, ChAgo-DM (Figure S1). ChAgo was loaded with 18 nt gDNA or gRNA followed by the addition of complementary 45-nt long ssDNA or RNA targets. After a 40-min incubation at 37 °C, the resulting products were separated on a 20% denaturing polyacrylamide gel. In reactions involving DNA targets and either gDNA or gRNA, ChAgo employed 5′-phosphorylated gDNA (5′P-gDNA) to cleave complementary ssDNA at a specific site between nucleotides 10 and 11 on the gDNA template, exhibiting a cleavage pattern consistent with that of previously characterized Ago proteins [19], leading to the production of a 34-nucleotide-long 5′-fragment of the DNA target (Figure 2b). In reactions containing an RNA target, ChAgo utilized 5′P-gDNA to cleave the complementary RNA target sequence between nucleotides 10 and 11 of the gRNA, generating a 34-nucleotide-long 5′-fragment of the RNA target (Figure 2b). No RNA cleavage was observed when using a 5′ hydroxylated DNA guide (5′OH-gDNA), whereas DNA cleavage was observed with the same guide (Figure 2b). The efficiency of RNA cleavage was lower compared to DNA cleavage. The catalytic tetrad in the PIWI domain was essential for ChAgo cleavage, and point mutations in this tetrad abolished ChAgo activity (Figure S2). In the absence of the ChAgo protein, no cleavage products were detected (Figure 2b).

### 2.3. Effects of Metal Ions on ChAgo Cleavage Activity

**Figure 3:**
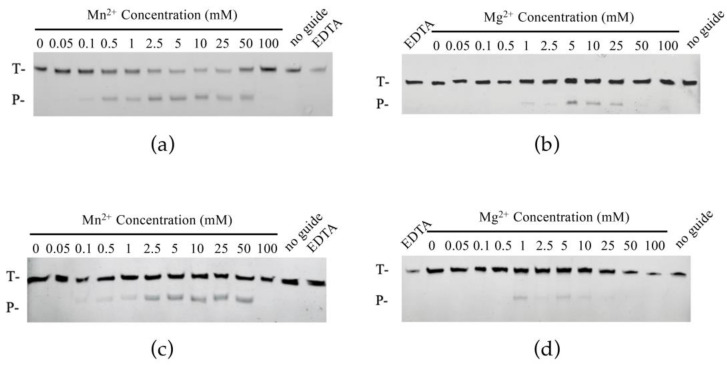
Effects of Mn^2+^ and Mg^2+^ concentration on ChAgo. (**a**) Effects of Mn^2+^ concentration on 5′P-gDNA mediated cleavage. (**b**) Effects of Mg^2+^ concentration on 5′P-gDNA mediated cleavage. (**c**) Effects of Mn^2+^ concentration on 5′OH-gDNA mediated cleavage. (**d**) Effects of Mg^2+^ concentration on 5′OH-gDNA mediated cleavage. T, ssDNA targets; P, cleavage products.

Given the necessity of divalent cations for Ago activity [3], we subsequently investigated their impact on DNA target cleavage. In the presence of different divalent metal ions (Mn^2+^, Mg^2+^, Ni^2+^, Co^2+^, Cu^2+^, Fe^2+^, Ca^2+^, and Zn^2+^), ChAgo was active with both Mn^2+^ and Mg^2+^ (Figure S3). Titration experiments with Mn^2+^ and Mg^2+^ ions revealed that ChAgo exhibited activity at Mn^2+^ concentrations of ≥0.1 mM, regardless of whether the guide was 5′P-gDNA or 5′OH-gDNA. Furthermore, ChAgo maintained equivalent activity within the range of 2.5 to 25 mM Mn^2+^ (Figure 3a,c). However, ChAgo was active at Mg^2+^ concentrations ≥ 1 mM (Figure 3b,d). Thus, ChAgo-mediated cleavage exhibited enhanced efficiency in the presence of Mn^2+^. Given that NaCl concentration may also influence cleavage activity, we assessed the efficiency of DNA cleavage across various NaCl concentrations. Notably, cleavage activity was significantly enhanced at lower NaCl concentrations (Figure S4).

### 2.4. Effects of Reaction Temperature and Guide Length on ChAgo Cleavage Activity

**Figure 4:**
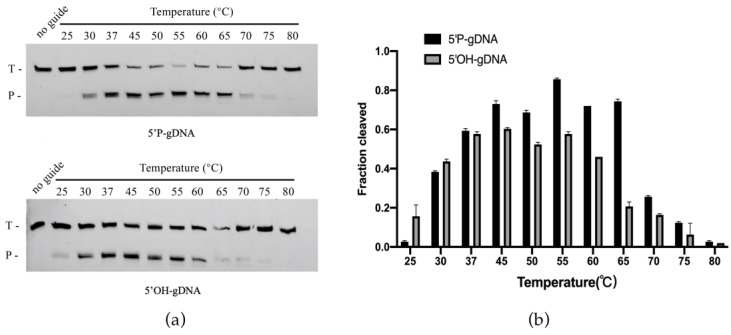
The impact of temperature on the activity of KmAgo. (**a**) Effects of temperature on 5′P-gDNA (**Upper panel**) and 5′OH-gDNA (**Lower panel**) mediated cleavage. T, ssDNA targets; P, cleavage products. (**b**) Quantification of cleavage efficiencies. Data are the mean ± SD from three independent measurements.

**Figure 5:**
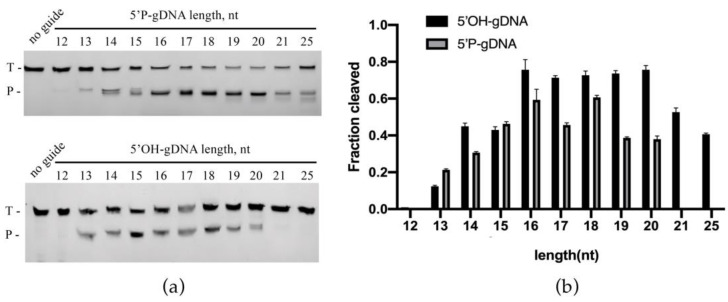
Effects of the guide length on ChAgo activity. (**a**) Cleavage assays utilizing 5′P-gDNA (**Upper panel**) and 5′OH-gDNA (**Lower panel**) of different lengths. T, ssDNA targets; P, cleavage products. (**b**) Quantification of cleavage efficiencies. Data are the mean ± SD from three independent measurements.

Analysis of temperature-dependent DNA cleavage activity showed that ChAgo bound to 5′P-gDNA exhibited robust cleavage activity between 37 °C and 65 °C, with decreased but detectable activity at 70–75 °C (Figure 4). For 5′OH-gDNA, the cleavage activity of ChAgo increased between 25 °C and 45 °C, but decreased when the temperature exceeded 55 °C (Figure 4). This suggests that interactions with the 5′ phosphate group are crucial for stabilizing the binary ChAgo–guide complex at higher temperatures. We evaluated the thermal stability of ChAgo and observed that the ChAgo–guide complex retained its activity following exposure to temperatures ranging from 37 °C to 60 °C for 30 min (Figure S5). We subsequently examined the impact of guide length by assessing a range of 5′P-gDNAs and 5′OH-gDNAs, spanning 12 to 25 nucleotides in length, which possessed identical sequences at their 5′ termini, ensuring a consistent predicted cleavage site across all guides. The ChAgo enzyme exhibited peak activity with 5′P-gDNAs ranging from 16 to 20 nucleotides and 5′OH-gDNAs ranging from 15 to 18 nucleotides, demonstrating reduced efficiency with guides outside this length range (Figure 5). Similar to KmAgo, the cleavage positions were shifted if shorter (12–15 nt) and longer (19–25 nt) 5′P-gDNAs were used [4].

### 2.5. Effects of Mismatches in the Guide-Target Duplex on the Cleavage Activity of ChAgo

**Figure 6:**
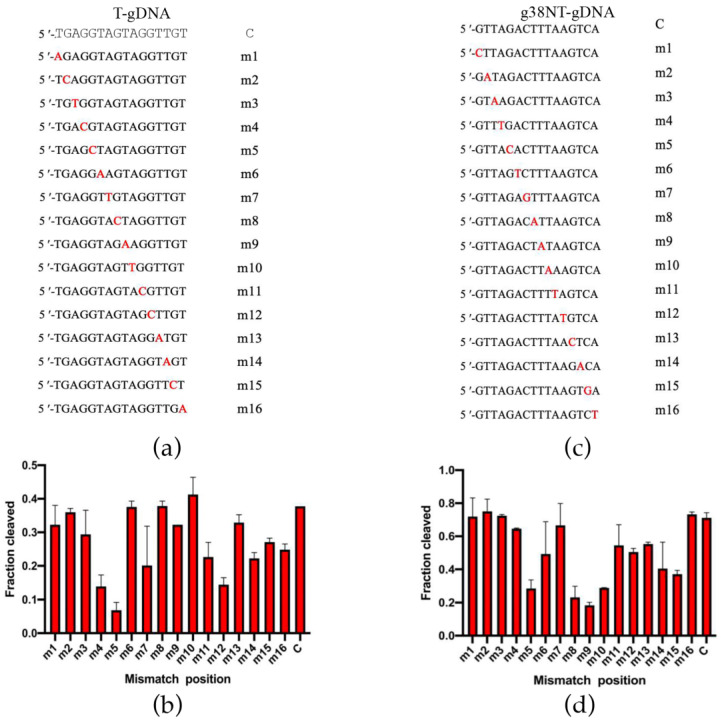
Effects of guide–target single mismatches on target cleavage. (**a**) Schematic diagram of let7-miRNA-based gDNA single nucleotide mismatch sites. Mismatched positions are indicated in red. (**b**) Effects of single nucleotide mismatches in the let7-miRNA-based 5′P-gDNA: tDNA duplex on the cleavage activity of ChAgo. (**c**) Schematic diagram of g38NT-based gDNA single nucleotide mismatch sites, mismatched positions are indicated in red. (**d**) Effects of single nucleotide mismatches in the g38NT-based 5′P-gDNA: tDNA duplex on the cleavage activity of ChAgo. Data are the mean ± SD from three independent measurements.

**Figure 7:**
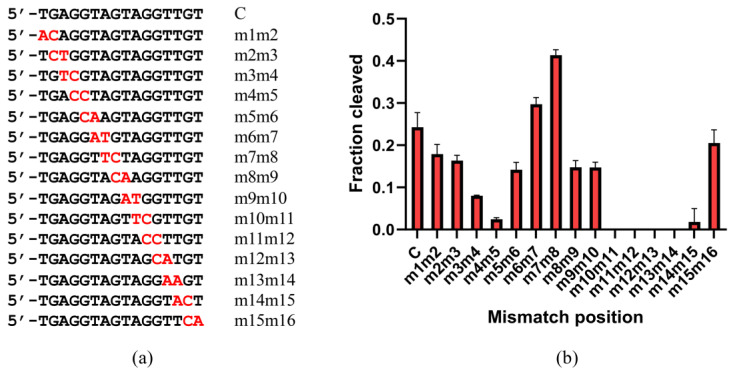
Effects of guide–target dual consecutive mismatches on target cleavage. (**a**) Schematic diagram of let7-miRNA-based gDNA double nucleotide mismatch sites. Mismatched positions are indicated in red. (**b**) Effects of double nucleotide mismatches in the let7-miRNA-based 5′P-gDNA: tDNA duplex on the cleavage activity of ChAgo. Data are the mean ± SD from three independent measurements.

Mismatches between the guide and target strands impair the cleavage activity of Ago proteins [31]. To assess the mismatch tolerance of ChAgo, we analyzed the impact of mismatches between the guide and target strands on its DNA cleavage activity. We designed a series of gDNAs, each containing a single-nucleotide mismatch at a specific position (Figure 6a, Table S1), and subjected them to DNA cleavage reactions with ChAgo. Mismatches at positions 4, 5, and 11–16 reduced the cleavage efficiency (Figure 6b). Notably, a significant reduction in cleavage efficiency was observed at position 5. To further investigate the impact of mismatches between gDNA and target strands, we systematically incorporated dinucleotide mismatches into the gDNA sequence (Figure 7a, Table S1). Although dinucleotide mismatches at positions 6 to 8 enhanced cleavage activity, mismatches at positions 3 to 5 and 10 to 15 significantly decreased cleavage efficiency (Figure 7b). To verify the low tolerance of ChAgo to mismatches, we synthesized another set of gDNAs with single nucleotide mismatches based on g38NT-gDNA used to characterize CbAgo activity [13] (Figure 6c), and tested the cleavage activity of ChAgo. Mismatches at positions 5 and 8–15 affected the cleavage efficiency, and a dramatic decrease was observed at positions 5 and 8–10 (Figure 6d). Thus, ChAgo has a low tolerance for mismatches between the guide and target strands.

## 3. Discussion

Here, we have described a novel mesophilic pAgo, ChAgo, isolated from a mesophilic bacterium and demonstrated that it can perform precise cleavage of ssDNA targets using short 5′P-gDNAs and 5′OH-gDNAs. ChAgo can cleave DNA when guided by 5′P-gDNA with lengths ranging from 14 to 25 nt. Our study revealed that ChAgo can cleave ssDNA requiring the participation of Mn^2+^ and Mg^2+^ ions. As a mesophilic enzyme, it functions at temperatures between 25 and 75 °C, showing peak activity at 55 °C.

We also found that ChAgo has a low tolerance for single-base mismatches, similar to most pAgo proteins [13,32]. Most of these mismatches likely result in minor perturbations or weak structural changes in ChAgo, without disrupting the formation of stable complexes with mismatched gDNA/target pairs. Notably, ChAgo exhibits significant variations in cleavage efficiency depending on the guide/target pair used. When the g38NT-gDNA/target pair was used, ChAgo exhibited higher cleavage efficiency compared to the T-gDNA/target pair. Moreover, for T-gDNA/target, mismatches at positions 4, 5, or 12 significantly reduce cleavage efficiency. However, for g38NT-gDNA/target, although the single-base mismatches at position 5 also led to a significant decrease in cleavage efficiency, positions 4 and 12 did not. Instead, positions 8–10 caused a significant decrease in cleavage efficiency. Therefore, ChAgo’s ability to distinguish single-base mismatches makes it a promising candidate for nucleic acid manipulation tools. Specifically, integrating ChAgo into diagnostic platforms for detecting gene mutations or pathogens could leverage its high specificity to ensure unprecedented accuracy in precise diagnosis. During application, it is crucial to first screen for sites with high cleavage efficiency and then identify and distinguish the positions of single-base mismatches based on the screened sequence.

## 4. Materials and Methods

### 4.1. Protein Expression and Purification

The nucleotide sequence of the ChAgo gene (WP_106544327; *Chroococcidiopsis* sp. CCALA 051) was codon-optimized for expression in *Escherichia coli*. The codon-optimized ChAgo gene was synthesized by Beijing Tsingke Biotech Co., Ltd., Beijing, China. and cloned into pET23a expression vector in a frame with the C-terminal His_6_ tag. The ChAgo double mutant (ChAgo-DM, D534A/D600A) was constructed by polymerase chain reaction (PCR)-mediated site-directed mutagenesis [33]. All cloned constructs were verified by DNA sequencing. *E. coli* BL21 (DE3) was used to express ChAgo and ChAgo-DM proteins. Cells were cultivated at 37 °C in Luria–Bertani (LB) broth supplemented with 100 μg/mL ampicillin and induced with 1 mM isopropyl-D-1-thiogalactopyranoside (IPTG) when the OD_600_ reached 0.6. The cells were then incubated at 18 °C with continuous shaking for 16 h to allow for protein expression. The harvested cells were stored at −80 °C for subsequent protein purification.

The cell pellet was resuspended in a lysis buffer consisting of 20 mM Tris-HCl (pH 7.4), 500 mM NaCl, and 10 mM imidazole, supplemented with 1 mM PMSF. The suspension was then disrupted using high-pressure homogenization (JNBIO-Mini Pro, JNBIO, Guangzhou, China, at 1000 bar). The lysate was clarified via centrifugation, and the resulting supernatant was loaded onto Ni-NTA agarose resin and rotated for 50 min. The beads were washed with Lysis Buffer of different concentrations. Fractions containing ChAgo or ChAgo-DM were concentrated via ultrafiltration utilizing an Amicon 50K filter unit (Millipore, Boston, MA, USA), placed in Storage Buffer [20 mM HEPES-NaOH (pH 7.4) and 500 mM NaCl], aliquoted, and flash-frozen in liquid nitrogen. Cell lysates and purified proteins were analyzed by 10% SDS-PAGE.

### 4.2. Phylogenetic Tree and Sequence Alignment of ChAgo

KmAgo was selected as the query and subjected to a BLASTp search using the NCBI database to identify Ago proteins with low sequence identity. Subsequently, the Ago protein sequences were aligned for phylogenetic analysis. The evolutionary relationship between ChAgo and several other well-studied Ago proteins was analyzed with the MEGA 11 [34].

### 4.3. Cleavage Assays

Cleavage assays were conducted using synthetic oligonucleotides (Table S1) under conditions described in a previous study [4]. An amount of 8 pmol of ChAgo was mixed with 4 pmol of guides and incubated for 10 min at 37 °C in a Reaction Buffer (10 mM HEPES-NaOH, pH 7.5, 100 mM NaCl, 5% glycerol) supplemented with 10 mM MnCl_2_. Subsequently, 2 pmol of the targets were added and incubated at 37°C for the specified time intervals. The reaction was terminated by combining the samples with an equal volume of 2× RNA loading dye (comprising 95% formamide, 18 mM EDTA, 0.025% SDS, and 0.025% bromophenol blue) and heating at 95 °C for 5 min. The cleavage products were separated by 20% denaturing PAGE. The gels were visualized using the GelDoc Go Imaging system (Bio-Rad, Hercules, CA, USA) and analyzed with ImageJ 1.53k (NIH, Bethesda, MD, USA) and Prism v8.3.1 (GraphPad, La Jolla, CA, USA).

To analyze the effect of divalent cations, 10 mM Mn^2+^, Mg^2+^, Ni^2+^, Co^2+^, Cu^2+^, Fe^2+^, Ca^2+^, Zn^2+^, or EDTA were used in the reactions, respectively. To determine the optimum concentration of Mg^2+^ and Mn^2+^, we performed gradient experiments with a final Mg^2+^ and Mn^2+^ concentration from 0 mM to 100 mM. To investigate the impact of reaction temperature, ChAgo loaded with guide RNA was first transferred to the specified temperatures, followed by the addition of targets and a 40-min incubation period. To examine the impact of guide length, we conducted experiments using a range of gDNAs with lengths varying from 12 to 30 nt. To assess the influence of guide–target duplex mismatches, we designed three sets of gDNAs, each containing mismatches at positions 1–16 (Table S1). All cleavage experiments were performed in triplicate.

### 4.4. Thermostability Assay

To analyze the thermostability of ChAgo, 800 nM ChAgo and 400 nM guide were preincubated at 37 °C for 10 min before being treated at 37, 44.1, 51.5, 55, 60, 64, or 70 °C for 30 min, respectively. Then, the ChAgo-guide duplex was used to cleave the 200 nM target at 37 °C for 15 min. The cleavage products were assayed by 20% denaturing PAGE.

## Data Availability

All relevant data of this study are presented. Additional data will be provided upon request.

## Supplementary Materials

The following supporting information can be downloaded at: https://www.mdpi.com/article/10.3390/ijms26031085/s1.
