# Supplementary material for: Characterization of Argonaute Nuclease from Mesophilic Bacterium Chroococcidiopsis
Source: Int J Mol Sci. 2025 Jan 27;26(3):1085. doi: 10.3390/ijms26031085 (PMC11817465; doi:10.3390/ijms26031085)
Supplement: Supplementary file 1 [file ijms-26-01085-s001.zip › ijms-3406206-supplementary.pdf]

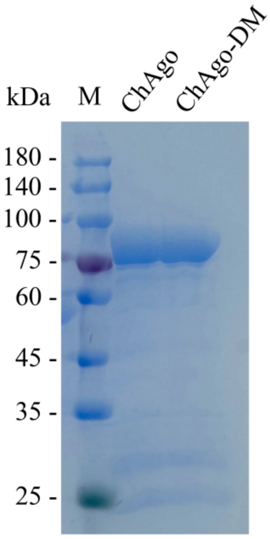

Figure S1. The purity of the purified ChAgo and ChAgo-DM was determined using SDS-PAGE. M, protein marker.

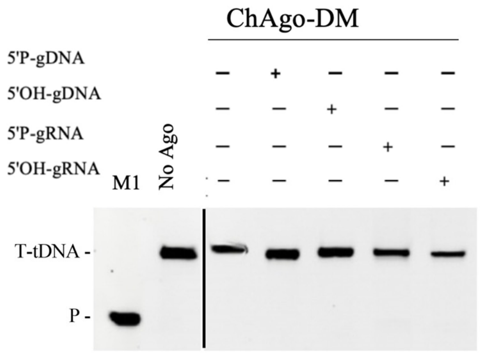

Figure S2. Cleavage activity assay of ChAgo-DM. The position of the cleavage products (P) is indicated on the left of the gels. M1, DNA marker. Representative gels from three independent measurements are shown.

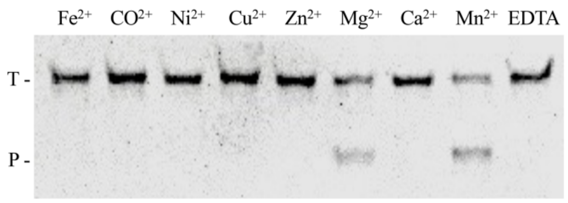

Figure S3. Effects of different cations on ChAgo.

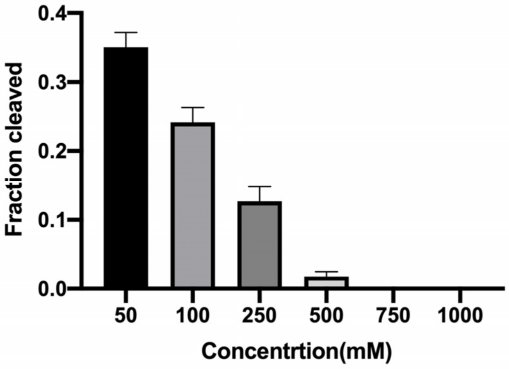

Figure S4. Effects of NaCl concentration on cleavage activity. Data are the mean ± SD from three independent measurements.

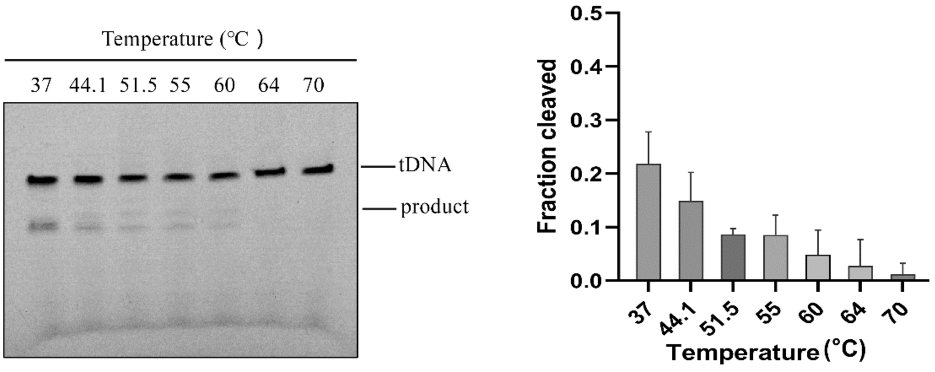

(a) (b)

Figure S5. Thermostability of ChAgo. (a) Effects of heat treatment temperature on the activity of ChAgo. Representative gels from three independent measurements are shown. (b) Quantification of cleavage efficiencies (the percentage of target cleavage). Data are the mean ± SD from three independent measurements.

Table S1. Oligonucleotide sequence used in the activity assays.

Oligonucleotide name	Sequence（5′-3′）	Description
T-gDNA	TGAGGTAGTAGGTTGTAT	Figure 2-6, 6a, 6b, S2-S4
gRNA	UGAGGUAGUAGGUUGUAU	Figure 2, S2
M1	FAM-AAACGACGGCCAGTGCCAAGCTTACTATACAACC	Figure 2, S2
M2	FAM-AAACGACGGCCAGUGCCAAGCUUACUAUACAACC	Figure 2
T-tDNA	FAM-AAACGACGGCCAGTGCCAAGCTTACTATACAACCTACTACCTCAT	Figure 2-5, 6a, 6b, S2-S4
U-tRNA	FAM-AAACGACGGCCAGUGCCAAGCUUACUAUACAACCUACUACCUCAU	Figure 2
T-gDNA-12 nt	TGAGGTAGTAGG	Figure 5
T-gDNA-13 nt	TGAGGTAGTAGGT	Figure 5
T-gDNA-14 nt	TGAGGTAGTAGGTT	Figure 5
T-gDNA-15 nt	TGAGGTAGTAGGTTG	Figure 5
T-gDNA-16 nt	TGAGGTAGTAGGTTGT	Figure 5
T-gDNA-17 nt	GAGGTAGTAGGTTGTA	Figure 5
T-gDNA-18 nt	TGAGGTAGTAGGTTGTAT	Figure 5
T-gDNA-19 nt	TGAGGTAGTAGGTTGTATA	Figure 5
T-gDNA-20 nt	TGAGGTAGTAGGTTGTATAG	Figure 5
T-gDNA-21 nt	TGAGGTAGTAGGTTGTATAGT	Figure 5
T-gDNA-25 nt	TGAGGTAGTAGGTTGTATAGTAAGC	Figure 5
gDNA_m1	AGAGGTAGTAGGTTGT	Figure 6a, b
gDNA_m2	AGAGGTAGTAGGTTGT	Figure 6a, b

gDNA_m3	TGTGGTAGTAGGTTGT	Figure 6a, b
gDNA_m4	TGACGTAGTAGGTTGT	Figure 6a, b
gDNA_m5	TGAGCTAGTAGGTTGT	Figure 6a, b
gDNA_m6	TGAGGAAGTAGGTTGT	Figure 6a, b
gDNA_m7	TGAGGTTGTAGGTTGT	Figure 6a, b
gDNA_m8	TGAGGTACTAGGTTGT	Figure 6a, b
gDNA_m9	TGAGGTAGAAGGTTGT	Figure 6a, b
gDNA_m10	TGAGGTAGTTGGTTGT	Figure 6a, b
gDNA_m11	TGAGGTAGTTCGTTGT	Figure 6a, b
gDNA_m12	TGAGGTAGTAGCTTGT	Figure 6a, b
gDNA_m13	TGAGGTAGTAGGATGT	Figure 6a, b
gDNA_m14	TGAGGTAGTAGGTAGT	Figure 6a, b
gDNA_m15	TGAGGTAGTAGGTTCT	Figure 6a, b
gDNA_m16	TGAGGTAGTAGGTTGA	Figure 6a, b
gDNA_m1m2	ACAGGTAGTAGGTTGT	Figure 7a, b
gDNA_m2m3	TCTGGTAGTAGGTTGT	Figure 7a, b
gDNA_m3m4	TGTCGTAGTAGGTTGT	Figure 7a, b
gDNA_m4m5	TGACCTAGTAGGTTGT	Figure 7a, b
gDNA_m5m6	TGAGCAAGTAGGTTGT	Figure 7a, b
gDNA_m6m7	TGAGGATGTAGGTTGT	Figure 7a, b
gDNA_m7m8	TGAGGTTCTAGGTTGT	Figure 7a, b
gDNA_m8m9	TGAGGTACAAGGTTGT	Figure 7a, b
gDNA_m9m10	TGAGGTAGATGGTTGT	Figure 7a, b
gDNA_m10m11	TGAGGTAGTTCGTTGT	Figure 7a, b
gDNA_m11m12	TGAGGTAGTACCTTGT	Figure 7a, b
gDNA_m12m13	TGAGGTAGTAGCATGT	Figure 7a, b
gDNA_m13m14	TGAGGTAGTAGGAAGT	Figure 7a, b

gDNA_m14m15	TGAGGTAGTAGGTACT	Figure 7a, b
gDNA_m15m16	TGAGGTAGTAGGTTCA	Figure 7a, b
g38NT_m1	CTTAGACTTTAAGTCA	Figure 6c, d
g38NT_m2	GATAGACTTTAAGTCA	Figure 6c, d
g38NT_m3	GTAAGACTTTAAGTCA	Figure 6c, d
g38NT_m4	GTTTGACTTTAAGTCA	Figure 6c, d
g38NT_m5	GTTACACTTTAAGTCA	Figure 6c, d
g38NT_m6	GTTAGTCTTTAAGTCA	Figure 6c, d
g38NT_m7	GTTAGAGTTTAAGTCA	Figure 6c, d
g38NT_m8	GTTAGACATTAAGTCA	Figure 6c, d
g38NT_m9	GTTAGACTATAAGTCA	Figure 6c, d
g38NT_m10	GTTAGACTTAAAGTCA	Figure 6c, d
g38NT_m11	GTTAGACTTTTAGTCA	Figure 6c, d
g38NT_m12	GTTAGACTTTATGTCA	Figure 6c, d
g38NT_m13	GTTAGACTTTAACTCA	Figure 6c, d
g38NT_m14	GTTAGACTTTAAGACA	Figure 6c, d
g38NT_m15	GTTAGACTTTAAGTGA	Figure 6c, d
g38NT_m16	GTTAGACTTTAAGTCT	Figure 6c, d
g38NT-gDNA	TTTAGACTTTAAGTCA	Figure 6c, d
g38NT-tDNA	FAM-TTTATCAAAAAGAGTATTGACTTAAAGTCT AAACTATAGGATACTTACAG	Figure 6c, d
